# Molecular Interaction, Chain Conformation, and Rheological Modification during Electrospinning of Hyaluronic Acid Aqueous Solution

**DOI:** 10.3390/membranes10090217

**Published:** 2020-08-31

**Authors:** Hao Chen, Xuhong Chen, Huiying Chen, Xin Liu, Junxing Li, Jun Luo, Aihua He, Charles C. Han, Ying Liu, Shanshan Xu

**Affiliations:** 1 Institute for Advanced Study, Shenzhen University, Shenzhen 518060, China; mason.hao.chan@gmail.com (H.C.); xuhongchen07@gmail.com (X.C.); chenhy997@gmail.com (H.C.); c.c.han@iccas.ac.cn (C.C.H.); 2 State Key Laboratory of Polymer Physics and Chemistry, Joint Laboratory of Polymer Science and Materials, Beijing National Laboratory for Molecular Sciences, Institute of Chemistry, Chinese Academy of Sciences, Beijing 100190, China; liuxin04@iccas.ac.cn (X.L.); lijunxing@iccas.ac.cn (J.L.); luoj@gtt.net.cn (J.L.); aihuahe@iccas.ac.cn (A.H.); 3 CAS Key Laboratory for Biomedical Effects of Nanomaterials and Nanosafety & CAS Center for Excellence in Nanoscience, National Center for Nanoscience and Technology of China, Beijing 100190, China; liuy@nanoctr.cn; 4 GBA Research Innovation Institute for Nanotechnology, Guangzhou 510700, China

**Keywords:** electrospinning, hyaluronic acid (HA), polyethylene oxide (PEO), chain entanglement, tissue engineering

## Abstract

Most of natural water-soluble polymers are difficult to electrospin due to their specific chain conformation in aqueous solution, which limits their applications. This study investigated the effects of polyethylene oxide (PEO) on the electrospinning of hyaluronic acid (HA) in HA/PEO aqueous solutions. The rheological properties of HA/PEO aqueous solutions showed polymer chain entanglement in HA was the essential factor affecting its electrospinnability. Wide-angle X-ray scattering and differential scanning calorimetry analyses of a PEO crystal showed different crystallization behavior of the PEO chain with different molecular weight, which indicates different interaction with HA. A schematic molecular model has been proposed to explain the effect of PEO on the chain conformation of HA along with the relationship between electrospinnability and chain entanglement. PEO with a relatively high molecular weight with limited crystal formation formed extensive chain entanglements with HA, while PEO with relatively low molecular weight weakened the interactions among HA chains. The findings of this study provide a wide perspective to better understand the electrospinning mechanisms of natural polyelectrolytes and usage in tissue engineering.

## 1. Introduction

Electrospinning has attracted considerable attention since the beginning of the 21st century as a simple and versatile method for manufacturing nanoscale structures in tissue engineering and biomedical field; for example, in neuronal and bone and tissue engineering [1,2,3,4]. Electrospinning is used to turn biodegradable polymers into fibrous products for application in tissue engineering scaffolds, wound dressings, drug delivery, and medical implants [5]. Natural polymers with a good biocompatibility usually possess poor electrospinning processability compared to synthetic polymers [6,7,8,9], especially from their aqueous solutions. The addition of salt solution can also increase the conductivity of the polymer solution, increases the surface charge of spinning jet, and makes spinning process more stable.

Generally, the properties of the spinning dope, including the polymer concentration, solvent volatility and elongational viscosity, play an important role in fiber formation during the electrospinning process [10,11]. A semi-empirical approach was used to demonstrate that chain entanglements resulting from the increased polymer concentration or molecular weight (Mw) can play a vital role in fiber formation during electrospinning, and stable fibers are formed when the polymer/good solvent system contains more than 2.5 entanglements per chain [12]. However, to obtain sufficient chain entanglements and a good electrospinning performance in polyelectrolyte solutions, the addition of screening ions or a significantly higher concentration of the polymer is required to overcome the electrostatic repulsive forces and the strong inter- and intra-molecular interactions of the polyelectrolyte chains [13].

**Scheme 1:**
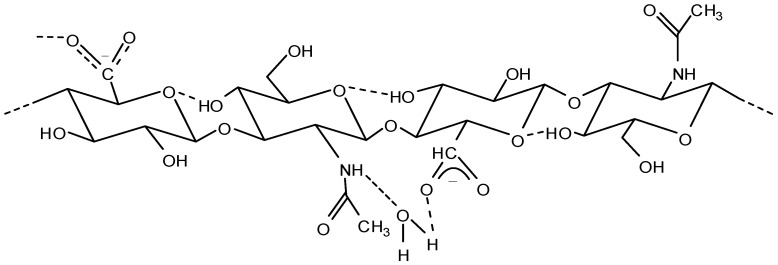
Secondary structure of hyaluronic acid (HA) in dimethylsulfoxide containing water. The dashed lines indicate hydrogen bonds.

As a naturally occurring linear polysaccharide with excellent biocompatibility, hyaluronic acid (HA) is the main component of the extracellular matrix [14]. HA chains contain two types of domains with different mobilities, and 55–70% of the structure of HA comprises the stiff part containing the cooperative structure. In an aqueous solution, HA molecules do not act as normal flexible polymers owing to the special structure and the extended hydrogen bonds in HA, as shown in Scheme 1 [15]. Generally, the electrospinning of HA in an aqueous solution is difficult. The unusually high viscosity and high surface tension of HA aqueous solutions are thought to be the key factors that hinder the electrospinning of HA aqueous solutions [15,16]. In a previous study, we found that fiber formation in HA solutions could be promoted by introducing dimethylformamide, which decreases the surface tension of the spinning solution and increases the elasticity of the HA solution [17].

In this study, we investigated the effect of the water-soluble polymer polyethylene oxide (PEO) on the electrospinnability of HA in aqueous solutions. The relationships between the solution properties and the electrospinnability were investigated by adding two types of polyethylene oxides (PEOs) with different molecular weights (Mws) to aqueous solutions of HA. The physical properties were fully characterized, including the conductivity, viscosity and surface tension, along with the rheological properties of the blended solutions. To understand the interactions between the HA and PEO polymer chains, the crystallization behavior of PEO in the HA/PEO blended fibers during electrospinning was characterized using polarized optical microscopy (POM), differential scanning calorimetry (DSC) and wide-angle X-ray scattering (WAXS). Based on the HA molecular structure and previous measurements, an entanglement model was established to explain the effect of PEO on the electrospinnability of the HA solutions. The characterizations and discussions in this study will broaden our understanding of the mechanism by which polyelectrolyte chains interact during the electrospinning process. Furthermore, the entanglement model provides another way to determine the factors that affect the electrospinnability of polymers.

## 2. Materials and Methods

### 2.1. Materials

Hyaluronic acid (sodium salt, Mw = 2,000,000) was purchased from Dali Co. (Nanning, China). Polyethylene oxide (PEO) with a Mw of 1,000,100 (PEO-100) was obtained from Sigma-Aldrich (St Louis, MO, USA). PEO with a Mw of 20,000 (PEO-2) was obtained from Beijing Chem. Co. (Beijing, China). All solvents were used without further purification.

### 2.2. Preparation of Spinning Solution

Transparent aqueous solutions of HA, PEO, and HA/PEO blends were prepared by dissolving specific amounts of the polymers in distilled water. The concentration of HA in the blended solutions was fixed at 1 w/v%. The concentrations of PEO-100 in the blended solutions were 0.5, 1 and 2 w/v%, and those of PEO-2 were 10, 20 and 40 w/v%. The concentrations of PEO-100 and PEO-2 in the pure aqueous solutions were 2 and 40 w/v%, respectively. The solutions were named with respect to the HA/PEO concentration ratio (Table 1). For example, the solution containing 1 w/v% HA and 2 w/v% PEO-100 was named “HA/PEO100 = 1:2”, and the pure HA solution was referred to as “HA.” The pure PEO-100 solution was named “PEO100”; thus, the fibrous product of PEO-100 was named “PEO100 fiber,” and its casting film was referred to as “PEO100 film.”

### 2.3. Electrospinning

The temperatures of all electrospinning solutions, spinnerets and the spinning environment were controlled at 30 ± 3 °C. Each electrospinning solution was placed into a 5-mL syringe with a capillary tip (inner diameter = 0.3 mm). A syringe pump was used to feed the polymer solution at a fixed feeding rate of 50 μL/min. A high-voltage power supply was employed to generate the electric field. The applied voltage was fixed at 20 kV, and the tip-to-collector distance was fixed at 15 cm. Owing to the strong water retention ability of HA, a copper frame with parallel copper wires was used to collect the fibers instead of aluminum foil. The electrospun HA/PEO fibrous membranes were placed in a vacuum oven at 30 °C for 1 h to remove the residual solvent.

### 2.4. Preparation of PEO Casting Films

Transparent aqueous solutions of PEO-100 and PEO-2 with concentrations of 2 and 40 w/v%, respectively, were prepared by dissolving specific amounts of the PEO powders in distilled water. The PEO solutions were dropped onto different pieces of glass. After natural evaporation at 30 °C for 24 h, the PEO casting films were placed into a vacuum oven at 30 °C for 1 h to remove the residual solvent.

### 2.5. Characterization

#### 2.5.1. Characterization of the HA and HA/PEO Blended Solutions

The conductivities and surface tensions of the HA-based solutions were measured using a conductivity meter (DDS-307A, Shanghai Rex Instruments, Shanghai, China) and a surface tension meter (DCAT 21, Dataphysics) using the Wilhelmy plate method at 40 °C, respectively. The shear viscosity and rheological properties (G’ and G’’) of the spinning solutions were measured at 30 °C using an Advanced Rheometric Expansion System (Ares, TA Instruments, New Castle, DE, USA). A Couette geometry of 17 × 16 mm was used in the measurements. For dynamic measurements, the frequency was controlled from 0.01 to 100 rad/s, and the strain was fixed at 1%. For constant measurements, the shear rate was increased from 0.01 to 100 s^−1^.

#### 2.5.2. Characterization of the HA/PEO Blended Fibers

The morphologies of the electrospun fibers were observed using scanning electron microscopy (SEM, HITACHI S-4300, HITACHI, Tokyo, Japan). WAXS analysis was performed under vacuum using an X-ray scattering instrument (Rigaku, The Woodlands, TX, USA) with Cu Kα radiation (λ = 0.15406 nm). The voltage and current of the generator (MicroMax-007 HF, Rigaku, The Woodlands, TX, USA) were 40 kV and 30 mA, respectively. The scattering patterns were calibrated in the q-space using an isotropic silver behenate powder standard, and the scattering length, q, ranged from 0.4 to 4.5 A^−1^. Thermogravimetry analysis (TGA) was performed using a PerkinElmer Pyris 1 TGA thermal analyzer. The pure HA fibers and pure PEO fibers were heated from 20 to 750 °C at a rate of 20 °C/min in a dynamic nitrogen atmosphere. Fourier transform infrared spectroscopy (FT-IR) spectra were performed using an infrared spectrometer (BRUKER TENSOR 27, Berlin, German) at 4 cm^−1^ resolution with 32 scans. A range of 4000–200 cm^−1^ was used for factor analysis.

To determine the effect of HA on the crystallization and melting behaviors of the PEO components, the HA/PEO blended fibers and the PEO casting films were analyzed by DSC using a Mettler DSC-822e calorimeter. The test samples (approximately 3 mg) were heated from 25 to 100 °C at a rate of 5 °C/min. The melting behavior of the HA/PEO100 = 1:1 sample was then measured under the following thermal program: (1) heated from 25 to 80 °C at a rate of 5 °C/min; (2) held at 80 °C for 5 min; (3) rapid cooling from 80 to 25 °C; (4) held at 25 °C for 10 min; (5) heated from 25 to 200 °C at a rate of 5 °C/min; (6) held at 200 °C for 5 min; (7) rapidly cooled from 200 to 25 °C; (8) held at 25 °C for 10 min; (9) heated from 25 to 250 °C at a rate of 5 °C/min; (10) held at 250 °C for 5 min; (11) rapidly cooled from 250 to 25 °C; (12) held at 25 °C for 10 min; and (13) heated from 25 to 80 °C at a heating rate of 5 °C/min. A plot of the thermal process was given to provide a better understanding of the results.

The melting process and isothermal crystallization process were observed using POM (Olympus BX51, Tokyo, Japan). The HA/PEO-2 blended fibers were placed on a hot stage (Linkam LTS350, Epsom, UK) and heated from 25 to 80 °C at a rate of 5 °C/min. The blended fibers were held at 80 °C for 5 min, rapidly cooled to 25 °C, and then held at 25 °C for 10 min to observe the recrystallization of PEO-2 in the blended fibers.

## 3. Results and Discussion

Although the HA used in this study possessed a high Mw, electrospinning of the HA aqueous solutions remained difficult owing to the rigid chain conformation of HA. To improve the electrospinnability of the HA and to further investigate the molecular interactions and rheological effect, two types of water-soluble PEO with different Mws were added to the HA solutions. Various characterization approaches were then applied to develop an intuitive image of the electrospinning process.

### 3.1. Electrospinning of the HA and HA/PEO Aqueous Solutions

Electrospinning of the pure HA aqueous solution (HA concentration = 1 w/v%) was attempted. The temperature of the environment was maintained at approximately 30 °C to increase the rate of evaporation of water. The electrospinning process was not continuous, and many droplets formed instead of a stable jet. No fibers were collected using the wire frame collector.

**Figure 1:**
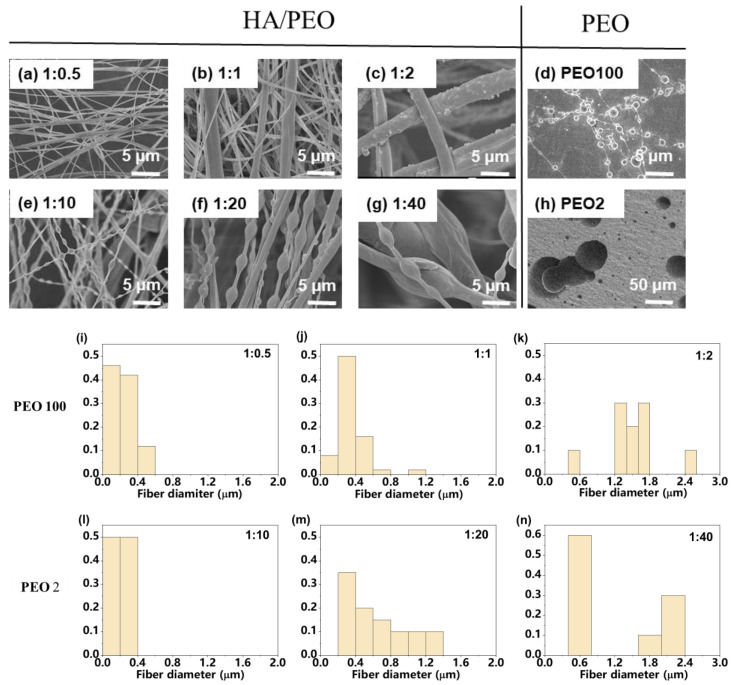
Scanning electron microscopy (SEM) images of the HA-based fibrous membranes and pure polyethylene oxide (PEO) fibers after electrospinning at 30 °C. The blended spinning solutions were (**a**) HA/PEO100 = 1:0.5; (**b**) HA/PEO100 = 1:1; (**c**) HA/PEO100 = 1:2; (**e**) HA/PEO2 = 1:10; (**f**) HA/PEO2 = 1:20; and (**g**) HA/PEO2 = 1:40. The pure PEO spinning solutions were: (**d**) PEO-100-0.5 w/v%; and (**h**) PEO-2-40 w/v%. Diameters distribution of HA-based fibrous membranes and pure PEO fibers: (**i**) HA/PEO100 = 1:0.5; (**j**) HA/PEO100 = 1:1; (**k**) HA/PEO100 = 1:2; (**l**) HA/PEO2 = 1:10; (**m**) HA/PEO2 = 1:20; and (**n**) HA/PEO2 = 1:40.

PEO aqueous solutions are known to possess a good electrospinnability if the Mw of PEO and the solution concentration are appropriate. When PEO-100 was added to the HA solution at a concentration of only 0.5 w/v%, a stable process with some jet disturbances was observed. As the PEO-100 concentration increased, the electrospinning process of the HA solution became more stable, and the fiber diameters increased significantly, as shown in Figure 1a–c. The electrospinning of pure PEO-2 was not successful; no fibers were obtained, even when the PEO-2 concentration reached 40 w/v%. This result indicates that the polymer chain of PEO-2 was too short to form sufficient entanglements, even at a high concentration of 40 w/v%. However, when PEO-2 was added into the HA aqueous solution at a concentration of 10 w/v%, a stable jet was formed, and bead-on-string fibers were obtained. As shown in Figure 1e–g, the bead-on-string structures were observed throughout the entire process.

### 3.2. Characterization of the HA Solutions and Their Electrospinnability

**Figure 2:**
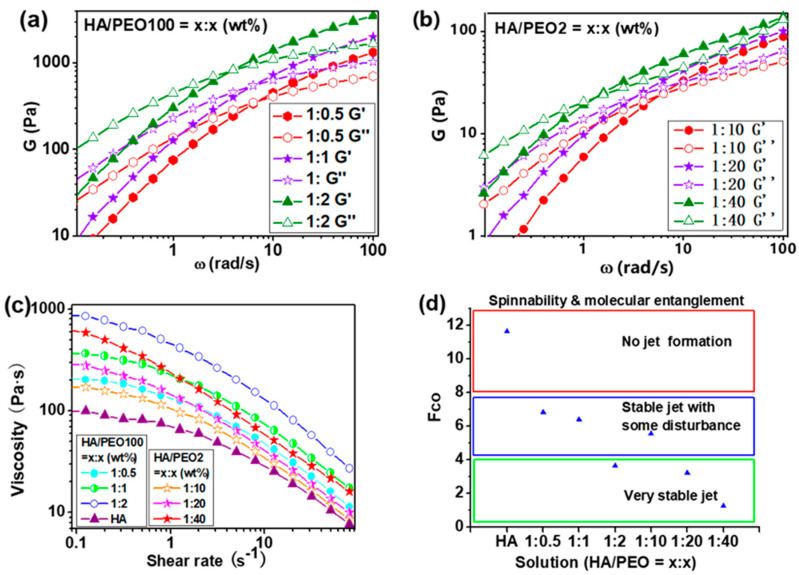
Rheological properties and shear viscosities of the HA/PEO blended solutions: G’ and G’’ of: (**a**) HA/PEO-100; and (**b**) HA/PEO-2 blended solutions. (**c**) Shear viscosities of HA and HA/PEO blended solutions as functions of the shear rate; and (**d**) *F_co_* versus electrospinnability.

The processing parameters did not noticeably change during the electrospinning of different solutions. Therefore, the properties of the dope solutions contributed to improving the electrospinning properties of HA. Increasing the content of PEO significantly increased the viscosity of the aqueous solution (Figure 2c) along with the fiber diameter (Figure 1 and Table 1). In addition, both the surface tension and conductivity of the blended solutions (Table 1) decreased upon the introduction of PEO. Thus, it seems that PEO, and particularly PEO-2 with a low Mw, acted as surfactants, and the neutral polymer chains significantly decreased the surface tension and conductivity.

The high surface tension and high viscosity are thought to be the main factors that hinder the electrospinning of HA aqueous solutions. However, the results shown in Table 1 and Figure 2c indicate that not all solutions with a low surface tension and low viscosity produced stable jets. Thus, in this system, neither the high viscosity nor high surface tension were found to be the essential factor preventing stable jet formation. Accordingly, rheological measurements were carried out to understand the effect of PEO on the chain entanglement and hydrodynamic behavior in the HA solution. As shown in Figure 2a and b, G” was larger than G’ at a low frequency in the HA/PEO blended solutions, indicating a viscous liquid character. Both G’ and G’’ increased with the increasing PEO concentration, indicating that both the viscosity and elasticity of the system increased with the increasing PEO concentration.

The frequency at the crossover point (*F_co_*) of G’ and G’’ indicates the degree of entanglement of the polymer chains [13,14]. The shift in *F_co_* towards a lower frequency reflects the formation of networks owing to physical crosslinking or enhanced chain entanglement [11,12]. Chain entanglement is an important parameter that can significantly influence fiber formation during polymer electrospinning. Therefore, *F_co_* was calculated from the curves of G’ and G’’ (Figure 2a,b). A high correlation was found between the electrospinning quality and *F_co_*; the electrospinning quality improved as the *F_co_* decreased (Figure 2d). When the PEO concentration increased, the *F_co_* decreased as the degree of chain entanglement increased, resulting in improved electrospinnability. In summary, the rheological results supported the conclusion that chain entanglement was the essential factor affecting the electrospinnability of the HA aqueous solution in this study.

### 3.3. Characterization of the HA/PEO Bicomponent Nanofibers

The above-mentioned results demonstrate that both PEO-100 and PEO-2 enhanced the chain entanglement in the HA solution. The PEO and HA were supposed to mix at the molecular level; thus, chain entanglements may occur on the molecular scale. This means that the HA molecules would affect the crystallization behavior of PEO. Therefore, to develop a deeper understanding of the interactions between the HA and PEO molecules with different chain lengths, WAXS, DSC and POM measurements were performed to characterize the crystallizability of PEO.

**Figure 3:**
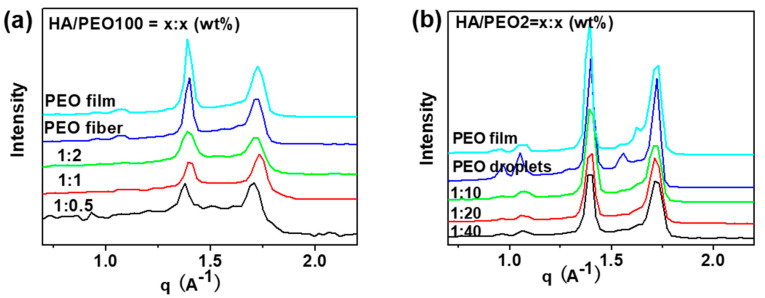
**Wide-angle X-ray scattering** (WAXS) patterns of the HA/PEO blended fibers and PEO casting films: (**a**) HA/PEO-100 blended fibers and PEO-100 fibers and casting film; and (**b**) HA/PEO-2 blended fibers and PEO-2 droplets and casting film.

As shown in Figure 3, all the WAXS patterns showed two strong peaks at q = 1.40 and 1.72 A^−1^, corresponding to the 120 and 112 reflections of crystalline PEO, respectively. This indicates that the crystalline structures of PEO were not significantly different from the HA/PEO blended fibrous samples and the pure PEO samples. However, Figure 3a and b reveal some differences between the blended and pure samples. The baseline spectra of the HA/PEO100 fibrous samples were rough and did not lie on the same horizontal line, which could all reflect the partial integrity of the crystals compared to the HA/PEO2 samples.

**Figure 4:**
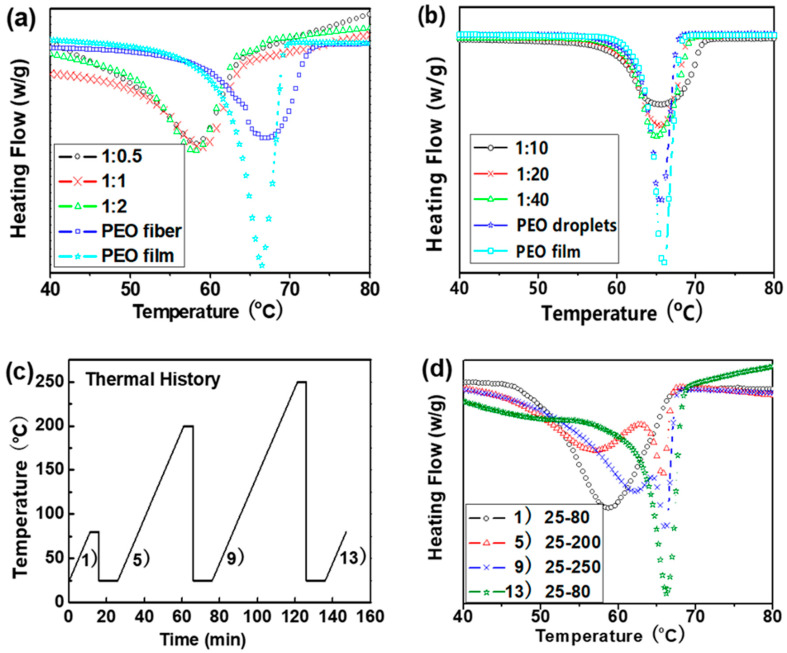
**Differential scanning calorimetry** (DSC) -determined melting behaviors of: (**a**) HA/PEO-100 blended fibers, PEO-100 fibers and the casting film and (**b**) HA/PEO-2 blended fibers, PEO-2 fibers and the casting film. Melting behavior of the HA/PEO100 = 1:1 blended fibers under a continuous thermal process: (**c**) thermal process; and (**d**) DSC pattern of some of the related processes.

In the DSC patterns (Figure 4a,b), the peaks for the PEO films are sharper and narrower than those of the electrospun samples. This indicates that the crystalline integrity of PEO molecules was lower in the electrospun samples than in the casting films. Thus, the electrospinning process had a negative effect on the crystallization of PEO. Moreover, the melting points of the HA/PEO-100 and HA/PEO-20 samples showed a variable shift to the left (to a lower temperature) compared to those of the pure PEO samples. The fast whipping and volatilization process during electrospinning might have a greater effect on the crystalline behavior of the high-Mw PEO compared to short-chain PEO.

The melting point (*T_m_*) and melting enthalpy (*ΔH*) of each sample were calculated from the DSC patterns (Table 2). The enthalpies of the electrospun samples were approximately 30 Jg^−1^ lower than those of the casting films. The *T_m_* values of the pure PEO samples were not significantly different. However, the Tm values of PEO-100 in the blended fibers were approximately 8 °C lower than those of the pure PEO-100 samples. This is attributed to the entanglements between the HA and PEO-100 molecular chains, which led to the confined crystallization of PEO. The lower Tm of PEO-100 compared to PEO-2 in the blended fibers indicates that the entanglement behavior between HA and PEO molecules differed based on the Mw of PEO. If the chain entanglement between HA and the high-Mw PEO occurs on the molecular scale as we hypothesized, the confined crystallization behavior of PEO should be acceptable. To confirm this, the melting behavior of the HA/PEO100 = 1:1 blended fibers was characterized under a continuous thermal process, as described in the Experimental section and shown in Figure 4c. 

Some of the DSC patterns from the heating process are shown in Figure 4d. The first DSC scan in the black circle shows a single, broad melting peak at approximately 58°C, which is the same as that observed for the HA/PEO (1:1) fibers shown in Figure 4a. After the first recrystallization, a new peak appeared close to 67 °C, which is similar to the melting point of pure PEO. The broad melting peak at about 58 °C was retained until the blended fibers were thermally treated at 250 °C, at which point the HA has already decomposed. These results indicate that the interactions between the HA and PEO-100 chains were not destroyed completely, even when the temperature exceeded the melting point of PEO-100. These strong interactions should improve the electrospinnability of the HA system.

### 3.4. Model Showing the Effect of PEO on HA Chain Conformation

**Figure 5:**
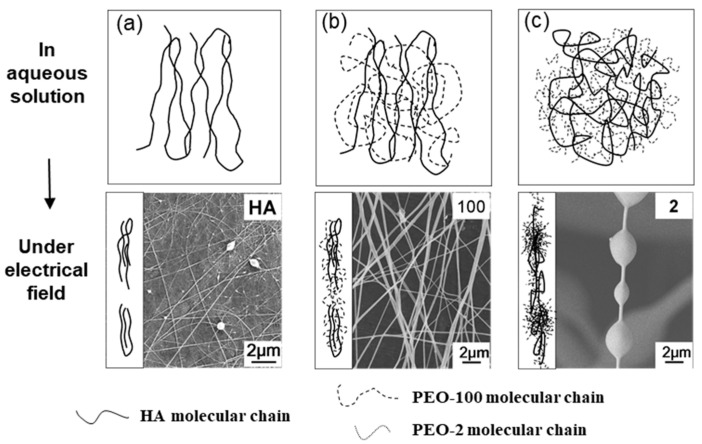
The chain entanglement model: (**a**) Pure HA, stiff polymer chains, only form few flections, and polymer chains will slip away when elongated by electric field; (**b**) HA/PEO-100 blends. Flexible PEO-100 chains pass through HA and entangle with other PEO molecules, and stabilize electric spinning process; (**c**) HA/PEO-2 blends. PEO-2, much smaller molecules, reduce the electrostatic repulsive forces and hydrogen bonding interaction, to make HA chains more flexible and form efficient entanglements.

Based on all of the above-mentioned characterization results, a model describing the effect of PEO on HA chain conformation was established. HA in the aqueous solution behaves as a stiff polymer. Therefore, the HA chains form entanglements, even at very low concentrations. As more than 50% of the HA molecular structure is stiff, the molecular entanglements among the HA chains are not the same as the entanglements formed in flexible chains. The proposed chain conformation of HA involves an overlap between two stiff polymer chains. Figure 5a shows a schematic diagram of a stiff HA chain with only a few flections. Although the stiff polymer chains were elongated under an electric field, they slipped away rather than hooking on to each other. This may explain why droplets with very few fibers were formed during the electrospinning of the pure HA aqueous solution.

PEO-100 is a linear and flexible polymer with very long chains. Even at a low concentration of 0.5 w/v%, the electrospinning process of the pure PEO solution was continuous, and a bead-on-string structure was formed, as shown in Figure 1d. This demonstrates that the entanglements of the PEO-100 chains were sufficient for electrospinning. It can be proposed that the flexible chains of PEO can pass through HA and entangle with other PEO molecules, as shown in Figure 5b. Hence, the PEO chain entanglement was not disrupted under the electric field, and HA/PEO bicomponent fibers were obtained. This model coincides with that of Shuang Wu’s [18], they indicated that the absence of superfine nanofibers was attributed to the size limit of high molecular weight PEO, which proved by continued increase of nanofibers diameter with the increase of PEO amounts.

The behavior of the low-Mw PEO-2 was significantly different from that of PEO-100. When 10 w/v% PEO-2 was added into the HA solution, the electrospinning process became stable. A concentration of 10 w/v% PEO-2 did not form sufficient entanglements. Even when the PEO-2 concentration was increased to 40 w/v%, the pure PEO-2 aqueous solution could not be electrospun, as shown in Figure 1h. This suggests that the stable jet formation might be attributed to the entanglements of the HA chains. The small-Mw PEO might form spatial obstructions in other polyelectrolyte molecules. 

In this study, the molecular chains of PEO-2 screened the electrostatic repulsive forces along the polymer backbones and weakened the hydrogen bonding interactions. These effects might have caused the HA molecular chains to become more flexible and form efficient entanglements, as shown in Figure 5c. Therefore, under an electric field, the HA chains hooked onto each other with PEO-2 coiled onto the HA. In this case, HA could be elongated into fibers, and PEO-2 was primarily present in the form of beads on the HA fibers. These beads did not disappear when the PEO concentration increased.

**Figure 6:**
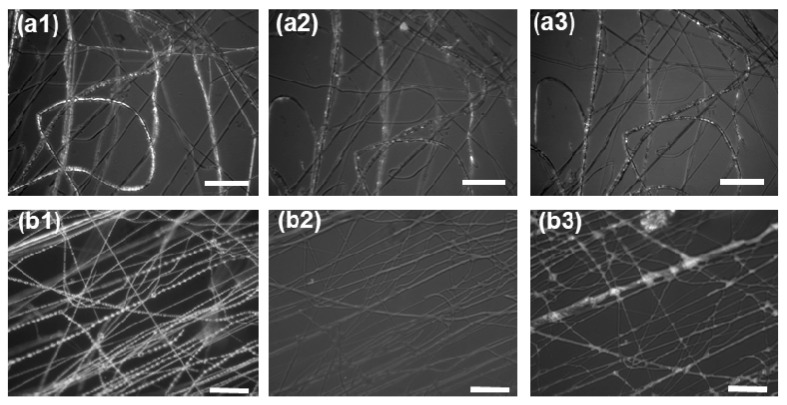
Polarized optical microscopy (POM) images of (a) HA/PEO100 = 1:2 and (b) HA/PEO2 = 1:20 films at different stages of heat processing: (**a1**,**b1**) 25 °C; (**a2**,**b2**) 80 °C for 5 min; and (**a3**,**b3**) 25 °C for 10 min. Scale bar = 5 μm.

The proposed model for the molecular entanglements was verified through an indirect experiment that was performed under heating and monitored using a phase-contrast microscope. The morphologies of the HA/PEO-100 blended fibers did not change significantly during heating. The brightness of the PEO crystals did not completely disappear upon heating for 5 min at 80 °C (Figure 6a); this temperature is approximately 10 °C higher than the melting point of pure PEO. This indicates that the entanglements between HA and PEO-100 were tight and effective, in agreement with the DSC results shown in Figure 4a. Thus, the HA chains limited the movements of the PEO-100 chains. However, the beads on the HA/PEO-2 fibers (Figure 6b) melted when the temperature exceeded the melting point of PEO-2, and the melting beads subsequently congregated into larger beads. The fibrous frame did not change throughout the entire process. These phenomena all indicate that HA chains were present along the fibers, and that the PEO-2 chains formed the beads. In contrast, HA and PEO-2 did not form effective entanglements owing to the short chain length of PEO-2. The blended solutions could be electrospun into beaded fibers, possibly because the addition of PEO-2 molecules improved the flexibility of the HA chains. This experiment validated the entanglement model and improved our understanding of the chain configurations and molecular movements of polyelectrolytes during electrospinning.

**Figure 7:**
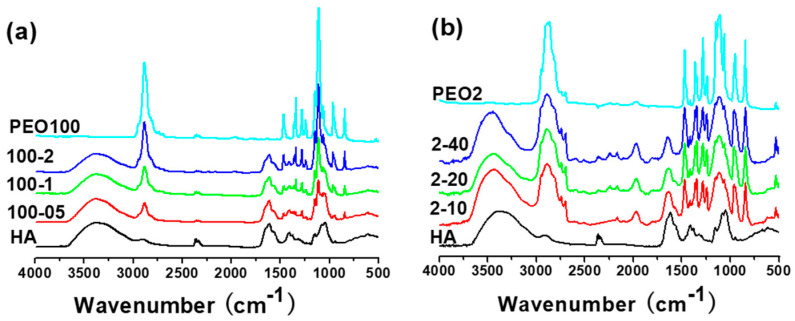
Fourier transform infrared spectroscopy (FT-IR) spectra of the HA/PEO blended electrospun membranes and HA and PEO casting films. (**a**) HA/PEO-100 blended membranes and PEO-100 casting film; and (**b**) HA/PEO-2 blended films and PEO-2 casting film.

The different interactions between the HA and PEO with different Mws were investigated using Fourier transform (FT)-Raman spectra, as shown in Figure 7. The patterns of the HA/PEO100 membranes did not show a significant difference from the pure HA film, indicating no strong bonding interactions. However, the shape of the peak at 1650 cm^−1^ (representing the C=O group) from the HA/PEO2 membranes changed significantly upon the addition of PEO2. Meanwhile, in contrast with the peak at 1650 cm^−1^ (representing the C=O group), the relative intensity of the peak at 3500 cm^−1^ (representing the hydrogen bonds) from the HA/PEO2 membrane increased with the increase in the PEO2 content. These results indicate that the interactions between the HA and PEO2 molecules are stronger than those to the PEO100 molecules. It should be noted that the end groups from PEO2 break the hydrogen bonds in the HA molecules instead of the ether groups. Therefore, water soluble PEO molecules improve the electrospinnablity of the HA aqueous solution by enhancing the entanglements of the system, but essentially through different interactions. PEO with a higher molecular weight enhances the entanglements through its own flexibility and entanglements, and PEO with a lower molecular weight enhances them by breaking the hydrogen bonds in the HA molecules.

According to Shanshan Xu’s research, the HA-based fibrous membranes present good cell viability, but bad cell proliferation [19]. This phenomenon is possibly related to the intrinsic nature of HA reported in previous literature [20]. Although HA possesses good biological compatibility and nontoxicity, HA is not suitable for cell attachment and proliferation. However, this feature of HA materials can be changed by increasing the gelatin content in HA/gelatin blend. Our further study will be published soon and provide an interesting result of the HA/gelatin bicomponent membrane with enriched gelatin content.

## 4. Conclusions

In this study, HA aqueous solutions containing the synthetic biopolymer PEO with different Mws were electrospun. The addition of PEO changed the chain conformation of the spinning solution and effectively improved the electrospinnability of the HA aqueous solution [21]. The Mw of the PEO affected the chain conformation of the blended solution.

In consideration of the stiff polymer chains of HA, a molecular entanglement model was established to explain the effect of PEO on the electrospinnability and fiber morphology. PEO with a relatively high Mw formed efficient chain entanglements and enhanced the chain entanglement in the blended solution. In contrast, PEO with a relatively low Mw did not form sufficient chain entanglements and disrupted the chain interactions among the HA chains by forming new interactions between the PEO and HA chains owing to the many end-hydroxyl groups present in the low Mw PEO. These findings provide a route for obtaining natural polymer nanofibers and are helpful for understanding the electrospinning processes of other polyelectrolytes.

## Figures and Tables

**Table 1:** Physical properties of the HA blended solutions and their electrospinnability.

Sample No. (HA/PEO = wt%:wt%)	Conductivity (ms/cm)	Surface Tension (mN/m)	Stability	Fiber Diameter (nm)
HA	1.20	71.3	-	No fiber
HA/PEO100 = 1:0.5	1.14	60.7	+	~200
HA/PEO100 = 1:1	1.11	59.0	+	200~500
HA/PEO100 = 1:2	1.02	54.9	++	~1200
HA/PEO2 = 1:10	0.70	51.8	+	~100
HA/PEO2 = 1:20	0.49	50.9	++	500~800
HA/PEO2 = 1:40	0.25	47.5	++	1000~3000

++ = very stable jet, continuous process; + = stable process with some jet disturbances; - = no jet formation observed.

**Table 2:** Detailed data from the DSC patterns of the HA/PEO blended fibers and PEO fibers and casting films.

Sample	*T_m_*/°C	Δ*H*/Jg^−1^	χ_c_ %
HA/PEO100 = 1:0.5	58.2	−109.4	51
HA/PEO100 = 1:1	59.0	−113.7	53
HA/PEO100 = 1:2	57.9	−122.2	57
PEO-100 fiber	66.7	−111.3	52
PEO-100 film	66.5	−147.8	69
HA/PEO2 = 1:10	65.6	−171.7	80
HA/PEO2 = 1:20	65.6	−161.3	75
HA/PEO2 = 1:40	65.0	−169.7	79
PEO-2 droplets	65.2	−168.7	79
PEO-2 film	66.0	−208.8	98
